# Who develops pandemic fatigue? Insights from Latent Class Analysis

**DOI:** 10.1371/journal.pone.0276791

**Published:** 2022-11-10

**Authors:** Steven Taylor, Geoffrey S. Rachor, Gordon J. G. Asmundson

**Affiliations:** 1 Department of Psychiatry, University of British Columbia, Vancouver, BC, Canada; 2 Department of Psychology, University of Regina, Regina, SK, Canada; Universitat de Valencia, SPAIN

## Abstract

According to the World Health Organization, pandemic fatigue poses a serious threat for managing COVID-19. Pandemic fatigue is characterized by progressive decline in adherence to social distancing (SDIS) guidelines, and is thought to be associated with pandemic-related emotional burnout. Little is known about the nature of pandemic fatigue; for example, it is unclear who is most likely to develop pandemic fatigue. We sought to evaluate this issue based on data from 5,812 American and Canadian adults recruited during the second year of the COVID-19 pandemic. Past-year decline in adherence to SDIS had a categorical latent structure according to Latent Class Analysis, consisting of a group adherent to SDIS (Class 1: 92% of the sample) and a group reporting a progressive decline in adherence to SDIS (i.e., pandemic fatigue; Class 2: 8% of the sample). Class 2, compared to Class 1, was associated with greater pandemic-related burnout, pessimism, and apathy about the COVID-19 pandemic. They also tended to be younger, perceived themselves to be more affluent, tended to have greater levels of narcissism, entitlement, and gregariousness, and were more likely to report having been previously infected with SARSCOV2, which they regarded as an exaggerated threat. People in Class 2 also self-reported higher levels of pandemic-related stress, anxiety, and depression, and described making active efforts at coping with SDIS restrictions, which they perceived as unnecessary and stressful. People in Class 1 generally reported that they engaged in SDIS for the benefit of themselves and their community, although 35% of this class also feared they would be publicly shamed if they did not comply with SDIS guidelines. The findings suggest that pandemic fatigue affects a substantial minority of people and even many SDIS-adherent people experience emotionally adverse effects (i.e., fear of being shamed). Implications for the future of SDIS are discussed.

## Introduction

According to the World Health Organization (WHO), pandemic fatigue during the current COVID-19 pandemic is a global problem that “poses a serious threat to efforts to control the spread of the virus” (p. 6) [1]. The cardinal feature of pandemic fatigue is a progressive decline in adherence to government guidelines for social distancing (SDIS), arising in the weeks or months in which SDIS and other pandemic-mitigation measures are in place. Declining adherence is hypothesized to be associated with pandemic-related burnout [1], which involves cynical or negative attitudes about the nature and origins of COVID-19 (e.g., belief that the threat of COVID-19 is exaggerated or a hoax), along with pessimism, apathy, and hopelessness about the efficacy of pandemic mitigation methods such as SDIS.

Pandemic fatigue is a chronic stress reaction in which the response to the stressor (i.e., decline in adherence to SDIS) perpetuates the stressor (i.e., facilitates the spread of pandemic infection) [2]. As pandemic fatigue sets in, people become increasingly lax about staying safe from infection. They increasingly disregard SDIS guidelines, such as by holding covert social gatherings or taking clandestine trips abroad [1–4]. A 2020 survey of over 7,000 American adults found that pandemic fatigue was characterized by progressively worsening adherence to the following SDIS guidelines: (a) remaining in residence except for essential activities or exercise, (b) having no close contact with non-household members, (c) not having visitors over to one’s home, and (d) avoiding eating at restaurants [5]. Similar findings have been reported in many countries [6]. Moreover, pandemic fatigue is not unique to COVID-19. During both COVID-19 and the 1918 Spanish flu pandemic, public cooperation with SDIS mandates deteriorated with successive waves of infection [7, 8]. For both COVID-19 and the Spanish flu, people in many communities objected to widespread closures and wanted to lift restrictions and resume normal life, despite active, widespread infection [9–11]. Pandemic fatigue is a problem even when vaccines are available since, as in the case of COVID-19, vaccines are far from completely effective, especially for the newer variants of the coronavirus causing COVID-19 [12].

Much remains to be learned about the nature of pandemic fatigue. It is unclear whether the decline in SDIS is unifactorial; that is, do all forms of SDIS progressively decline over time or are some forms of SDIS more likely to be adhered to than others? If non-adherence to SDIS is unifactorial, then the question arises as to whether this factor has a dimensional or categorical structure. That is, pandemic fatigue might be a matter of degree or, alternatively, there might be distinct types of people, such as those who generally adhere to SDIS versus those who become progressively non-adherent. According to the WHO, identifying at-risk groups for non-adherence can help guide efforts at reinvigorating communities to follow SDIS protocols [1]. Research is needed to identify which group or groups are at greatest risk for deteriorating adherence to SDIS.

Declining motivation to adhere to SDIS may be due to range of factors including decreases in perceived risk of COVID-19 as people become habituated to the viral threat. Other factors potentially contributing to declining adherence include accumulating costs or hardships associated with SDIS, such as growing economic losses from having remain at home, difficulties working or studying from home, and social isolation [1]. Also, non-adherence to SDIS has been found to be associated with the perception that social distancing interventions are unnecessary and ineffective [13], with younger age [14–17], greater perceived personal affluence [18], and lower trust in government [18]. Gender findings have been mixed, with some studies finding that non-adherence is more prevalent in men [14, 18] and others reporting greater prevalence in women [19]. People who disregard SDIS are more likely to have been infected with the coronavirus causing COVID-19 [20]. If the person’s COVID-19 symptoms are mild, then this might reinforce beliefs that the COVID-19 threat is exaggerated, thereby amplifying non-adherence.

Research is needed to further investigate how attitudinal, emotional, and personality variables are associated with declining adherence to SDIS. Although declining adherence is hypothesized to be related to pandemic-related burnout [1], this remains to be empirically established. If SDIS is distressing and isolating, then it is expected that anxiety, depression, and loneliness would also be associated with declining adherence to SDIS guidelines. People who are especially distressed might violate SDIS guidelines in pursuit of socially rewarding activities, such as attending social gatherings.

Adherence to SDIS guidelines requires individuals to put the needs of the community (e.g., the need for SDIS) ahead of their personal desires (e.g., desires to travel on holidays or go to social gatherings). Declining adherence to SDIS might be especially likely for people with high scores on particular personality traits, such as those who have a strong need to socialize with others (e.g., high levels of extraversion) and who value their own needs and desires above those of the community (e.g., high levels of narcissism or psychological entitlement). Accordingly, a deeper understanding of the nature of pandemic fatigue might be attained by investigating the relationship between declining adherence to SDIS and personality traits, particularly extraversion, narcissism, and psychological entitlement.

Narcissism and psychological entitlement are related but distinguishable constructs. Entitlement refers to a stable and pervasive sense that one deserves more compared to other people [21]. Narcissism is a broader construct involving self-absorption, grandiosity, arrogance, and a sense of entitlement [22]. Thus, entitlement can be a component of narcissism but high levels of entitlement can also occur in the absence of narcissism; that is, a person can feel entitled to special treatment without necessarily having an inflated sense of self-worth.

The primary aims of the present study were to (a) investigate the structure (i.e., dimensional vs. categorical) of declining adherence to pandemic-related SDIS guidelines, based on data collected during the COVID-19 pandemic, and (b) to identify the demographic, attitudinal, emotional, and personality characteristics of people most likely to become progressively non-adherent to SDIS. A further exploratory aim was to investigate the stated reasons as to why people adhered to SDIS guidelines, since this provides information about motivations for adherence. At the time of conducting the study, social distancing was in place along with closures or restricted operations of restaurants, and restrictions against travel and against large social gatherings.

## Materials and methods

### Sample

The sample consisted of 5,812 adults (age ≥18 years) from the U.S. (*n* = 2,968) and Canada (*n* = 2,844) who were recruited as part of the COVID Stress Study [23, 24], which is a broad investigation into the psychology of COVID-19. The mean age of the sample was 49 years (SD = 17 years, range 18–92 years). About half the sample (52%) were employed full- or part-time, most (78%) had completed full or partial college, and 57% were female. Most (64%) were White, with the remainder being Asian (13%), African American/Black (11%), Latino/Hispanic (4%), or other (7%). A total of 4% of the sample reported that they were healthcare workers and 7% stated that they had been diagnosed with COVID-19 by a healthcare worker. A third (32%) of respondents reported that they had been partially or fully vaccinated against the novel coronavirus at the time of the study, and 38% reported that they had a preexisting (pre-COVID-19) general medical condition. A total of 23% of respondents reported that they had a recent (past year) history of a mental health problems, predominantly mood or anxiety symptoms, and 71% believed that COVID-19 had harmed their mental health.

### Measures

#### Overview

With the exception of the single-item measures of socioeconomic status and political conservativism, as discussed later, all measures were multi-item instruments, as listed in Table 1. Table 1 shows the internal consistency reliabilities for the multi-item scales. McDonald’s ω total [25], which is a commonly used alternative to Cronbach’s α, was used as the measure of reliability. McDonald’s ω was used instead of Cronbach’s α because the latter tends to underestimate reliability [26]. Values of ω are interpreted in the same way as α; that is, values in the range of 0.70–0.80 indicate acceptable reliability, 0.80–0.90 are good, and values greater than 0.90 are excellent. Table 1 shows that all scales had at least acceptable reliability, and almost all (93%) had good-to-excellent reliability. Further details of these scales are as follows, organized according to the domains listed in Table 1.

**Table 1:** Internal consistency reliability coefficient (ω total) for multi-item scales.

Scale	Domain	No. items	ω total
**Changes in adherence to social distancing**	SDIS	10	0.87
**Disregard for social distancing, current**	SDIS	4	0.90
**Reasons for social distancing, current**	SDIS	8	0.86
**Agreeableness**	Personality	2	0.76
**Conscientiousness**	Personality	2	0.82
**Extraversion**	Personality	2	0.84
**Narcissism**	Personality	7	0.89
**Negative emotionality**	Personality	2	0.87
**Openness to experience**	Personality	2	0.76
**Psychological entitlement**	Personality	12	0.90
**Sociability**	Personality	5	0.90
**COVID-19 apathy**	Burnout	7	0.89
**COVID-19 blame**	Burnout	8	0.88
**COVID-19 burnout**	Burnout	12	0.96
**COVID-19 threat exaggerated**	Burnout	3	0.90
**COVID-19 pessimism**	Burnout	16	0.93
**Anxiety**	Emotion	7	0.96
**Depression**	Emotion	9	0.94
**Loneliness**	Emotion	3	0.91
**Distrust in government**	Attitude	14	0.93
**Belief in COVID-19 conspiracy theories**	Attitude	4	0.92
**Belief in robust personal health**	Attitude	3	0.91
**COVID-19 anti-vaccination attitudes, current**	Attitude	12	0.93
**Facemask non-adherence, current**	Attitude	3	0.86
**Coping with COVID-19**	Coping	38	0.94
**Stressors associated with COVID-19**	Stressors	22	0.95

Note: Burnout includes both attitude and emotion variables. SDIS = social distancing.

#### Social distancing scales

Past-year changes in adherence to SDIS were assessed by the 10-item face-valid SDIS Scale, developed for the purpose of the present study. The items, listed in Table 2, were each rated on a 3-point scale (1 = more often, 2 = no change, 3 = less often), meaning that higher scores were associated with a greater past-year deterioration in adherence to SDIS.

**Table 2:** Endorsement and factor loadings for items assessing changes in adherence to social distancing over the past year (total sample; *N* = 5,812).

Item	% less often	% no change	% more often*	Factor loading
**Close contact (within 6 feet) with people who don’t live with me**	44	45	12	0.50
**Go out to socialize with friends, family, or relatives**	54	37	9	0.66
**Have friends, neighbors, or relatives to my home**	48	44	8	0.64
**Socialize outside of my social bubble**	48	46	6	0.62
**Go to gatherings of more than 6 people**	46	49	6	0.65
**Have contact with people who could be high-risk for COVID-19**	39	57	4	0.50
**Go to crowded public places where social distancing is not possible**	45	51	4	0.60
**Go to a crowded bar, club, restaurant, or cafe**	42	55	4	0.57
**Travel outside of my state or province**	42	55	4	0.50
**Go to crowded church services**	31	66	3	0.50

*Indicates progressive decline in adherence to social distancing (SDIS).

Current disregard for social distancing (as distinct from past-year changes in SDIS) was measured by a 3-item face-valid scale developed and evaluated in our previous research [23, 24, 27]. Items on these measures were rated on a 5-point scale (0 = strongly disagree, 4 = strongly agree). Higher scores indicate greater non-adherence to SDIS. This scale had good levels of reliability and validity [27] (and see Table 1).

For people who adhered to SDIS, their reasons for adherence were assessed by an 8-item face-valid scale in which respondents rated their strength of agreement with each item. Those items, rated on a 3-point scale (1 = disagree, 2 = unsure, 3 = agree), appear in Table 3.

**Table 3:** Reasons for adhering to social distancing guidelines (Class 1 participants; *n* = 5,326).

Reason	% disagree	% unsure	% agree
**It keeps me safe from infection**	6	7	87
**It helps my community stay safe**	5	9	86
**It keeps my loved ones safe from infection**	6	9	86
**It helps my country overcome the pandemic**	6	11	83
**My government tells me to do it**	14	21	65
**People would disapprove of me if I didn’t engage in social distancing**	22	27	51
**I could get a fine if I didn’t do it**	38	27	36
**I would be publicly shamed if I didn’t do it**	36	29	35

#### Personality scales

A broad assessment of personality traits was conducted using the Ten Item Personality Inventory (TIPI) [28], which is a 5-scale measure of the Big 5 personality dimensions of extraversion, agreeableness, conscientiousness, negative emotionality, and openness to experience (2 items per scale describing a personality trait). For each item, respondents rated their agreement with each item in terms of whether it described their personality (e.g., “sympathetic, warm”) on a 7-point scale (1 = disagree strongly, 7 = agree strongly). Despite being a very brief measure, the TIPI has performed well on various indices of reliability and validity [28–30]. In the present study, the TIPI scales had acceptable-to-good levels of reliability (see Table 1).

Narcissism was measured by a 7-item scale from a larger, multi-scale battery known as the Short Dark Tetrad [31]. For the narcissism scale, respondents rated their strength of agreement on a 5-point scale (e.g., “I have some exceptional qualities”; 1 = strongly disagree, 5 = strongly agree). This scale has good reliability and validity [31] (also see Table 1).

Psychological entitlement was assessed using a 12-item version of the Psychological Entitlement Scale [21], in which participants rated, on a 7-point scale, the extent to which the respondent believed that he or she was entitled to special treatment in various aspects of life (e.g., “I honestly feel I’m just more deserving than others”; 1 = strongly disagree, 7 = strongly agree). Although psychological entitlement is related to narcissism (*r* = 0.44 in the present study), the two constructs are distinguishable in that entitlement entails beliefs in deserving special treatment without necessarily entailing, as in narcissism, an inflated sense of self-worth. The scale has good reliability and validity [21] (see also Table 1).

Sociability was assessed using the Sociability Scale [32], in which respondents rated their agreement on five statements (e.g., “I find people more stimulating than anything else”; 1 = strongly disagree, 5 = strongly agree). The scale has sound psychometric properties [32] (see also Table 1).

#### Burnout scales

Measures of COVID-19-related burnout were based on the Burnout Measure [33], which is a psychometrically sound measure of burnout. Five burnout-related scales were administered; COVID-19 related burnout (measuring emotional aspects of burnout), apathy, blame, pessimism, and belief that the coronavirus threat is exaggerated. All had good reliability (Table 1). For the COVID-19-related burnout scale, respondents were presented with 12 adjective statements (e.g., irritable, frustrated, emotionally exhausted) and were asked, “When you think about COVID-19, how often do you feel the following?” Statements were rated on a 5-point scale (1 = never, 5 = always).

COVID-19-related apathy was measured by 7 statements, each rated on a 5-point scale (e.g., “Regardless of what we do, almost everyone will get COVID-19”; 1 = strongly disagree, 5 = strongly agree). This rating scale was also used in the COVID-19-related blame and pessimism scales. Blame was assessed by 8 items (e.g., “People in my community are to blame for the spread of COVID-19”). COVID-19-related pessimism was assessed in a similar manner with 16 items (e.g., “There is nothing I can do to keep myself safe from COVID-19”).

Belief that the threat of COVID-19 has been exaggerated was measured by a 3-item scale developed and evaluated in our previous research [23, 24, 27]. Items were rated on a 5-point scale (0 = strongly disagree, 4 = strongly agree). This face-valid scale has good levels of reliability and validity [27] (and see Table 1).

#### Emotion scales

General anxiety and depression over the past week were assessed, respectively, by the GAD-7 [34] and PHQ-9 [35]. Both scales have good psychometric properties [34, 35] (also see Table 1). The tendency to feel lonely was measured using the Loneliness Scale [36] in which items assessing loneliness (e.g., “How often do you feel left out?”) are rated on a 3-point scale (1 = hardly ever, 3 = often). The scale has good psychometric properties [36] (also see Table 1).

#### Stress and coping scales

COVID-19 related stressors were assessed using a 22-item face-valid scale based on our previous research [24] in which respondents rated the frequency (1 = never, 5 = often) of various stressors experienced during COVID-19 (e.g., difficulty working from home, isolation, crowding at home, difficulty caring for loved ones). Despite covering a range of different, commonly occurring stressors during COVID-19 [24], the 22-item scale had excellent internal consistency (see Table 1).

Coping with COVID-19-related stress was assessed with a 38-item, face-valid scale [24] that assessed the frequency of use (1 = never, 5 = very often) of a range of different coping strategies (e.g., exercising, reading novels, talking with a trusted friend). The reliability of the scale was excellent (see Table 1).

#### Attitudinal measures

Belief that one has robust personal health against COVID-19, and belief in COVID-19 related conspiracy theories, were each assessed by a 3-item scale with items rated on a 5-point scale (0 = strongly disagree, 4 = strongly agree). These face-valid scales have good levels of reliability and validity [27] (and see Table 1). Distrust in government for managing the COVID-19 pandemic was assessed using a 14-item face-valid measure developed for the purpose of the present study. For each item, participants rated their agreement on a 5-point scale for statements such as “My government has allowed its citizens to be financially ruined by the pandemic” (1 = strongly disagree, 5 = strongly agree). The reliability of the scale was excellent (see Table 1).

Current anti-vaccination attitudes toward COVID-19 vaccines were measured using the Vaccination Attitudes Examination Scale [37], adapted to assess vaccination attitudes specific to COVID-19 [38]. The 12 items in this scale, each rated on a 6-point scale (0 = strongly disagree, 5 = strongly agree), assess mistrust of vaccine benefit, worries over unforeseen future effects of the vaccine, concerns about commercial profiteering from the vaccine, and preference for natural immunity. The scale has good levels of reliability and validity [37, 38] (also see Table 1).

Current non-adherence for wearing facemasks was assessed by a 3-item face-valid scale, in which respondents were asked to rate the frequency in which they intentionally refrained from wearing a mask in public places (e.g., in stores or on public transit). Items were rated on a 1–6 scale (1 = never, 6 = more than once a day).

#### Other measures

Respondents completed a short questionnaire assessing demographic information along with details regarding COVID-19, including whether they had received one or more doses of a COVID-19 vaccine, whether they had developed COVID-19, and whether they believed they COVID-19 had harmed their mental health. Each respondent’s perceived socioeconomic status, in relation to people in their country, was assessed by the MacArthur Scale of Subjective Social Status [39]. Respondents are asked to rate themselves on this single-item scale by choosing a number on a 1–10 scale that best represented their socioeconomic standing in relation to other people in their country. Higher scores corresponded to greater perceived socioeconomic status. The scale has been shown to perform well on various indices of reliability and validity [39–41]. Political conservatism was assessed with a face-valid 7-point scale developed in our previous research [42], in which respondents answer the following item, “In general, how would you describe your political views?” (1 = very liberal, 7 = very conservative).

### Data collection procedures

Data were collected from March 24 to May 4, 2021, at which time social distancing restrictions were implemented throughout the U.S. and Canada. The sample was obtained using an internet-based self-report survey delivered in English by Qualtrics, a commercial survey sampling and administration company. Qualtrics solicited the present sample, for which no data have yet been reported on or published, as part of our ongoing research program [23, 24, 42]. Qualtrics maintains a pool of potential participants who have agreed to be contacted in order to respond to surveys. Qualtrics selected and contacted participants to meet sampling quotas to approximate general population demographics, based on age, gender, ethnicity, socioeconomic status, and geographic region within each country. The demographic composition of the sample approximated census-derived data of U.S. and Canadian adults (i.e., excluding children and adolescents), where, for example, the mean age averaged across countries is 50 years and 67% white. The sample departed from census data in that females were over-represented (57%) as compared to census data (51%). However, gender was not substantively associated with any of the variables in this study; that is, effect sizes for gender were smaller than what is conventionally regarded as “small” effect sizes (see below).

All respondents provided written informed consent prior to completing the survey. The research described in this article was approved by the Research Ethics Board of the University of Regina (REB# 2020–043). Filters were used to eliminate data from careless responders. Embedded in the assessment battery were four attention-check items (e.g., “This is an attention check, please select Strongly Agree”; “For our research, it is really important that you paid attention while responding to our survey. How attentive were you when responding?”: “Very Inattentive” to “Very Attentive”). Participants were included only if they provided correct responses to three or more of the four attention checks (e.g., “Strongly agree” or “Very attentive”), indicating that they were sufficiently attentive. In addition, at the end of the assessment battery, participants were asked to indicate whether, in their honest opinion, their data should be used. Those who responded “no” were excluded from data analysis, regardless of their score on the attention-check items.

### Statistical procedures

Exploratory factor analysis using robust Maximum-Likelihood and Parallel Analysis were used to determine the number of factors of the SDIS scale. This was followed by Latent Class Analyses, also using robust Maximum-Likelihood, to determine whether the factor was dimensional or categorical in nature. Analyses were conducted using SPSS 27.0 and Mplus [43]. In the Latent Class Analyses, models consisting of increasing numbers of classes were evaluated (e.g., 1 vs 2 classes, 2 vs 3 classes) until the best‐fitting model was identified, as determined by four goodness‐of‐fit indices: Akaike Information Criterion, Bayesian Information Criterion, sample‐size adjusted Bayesian Information Criterion, and the Bootstrap Likelihood Ratio Test. For the first three fit indices, the best‐fitting model has the lowest value on these indices. For the Bootstrap Likelihood Ratio Test, the best fitting model is a model consisting of N classes, which has a significantly better (*p*<0.01) fit than a model consisting of *N*−1 classes, and is not significantly different from a model consisting of *N*+1 classes. The resulting number of classes were then compared on a range of emotion-related and other variables.

Given the number of analyses reported in this article, the α level was set at 0.01 instead of 0.05. This adjustment corrects for inflated Type I error without unduly inflating Type II error with a more stringent correction, such as a Bonferroni correction. Given the large sample size, substantively trivial effect sizes would be statistically significant (e.g., for *r* = 0.05, *p* < .001). Accordingly, to facilitate the interpretation of correlations, we used Cohen’s criteria [44] to classify effect sizes as small, medium, or large. Effect sizes were either Cohen’s *d* for pairs of variables in which one or both variables were continuous, or Cramér’s *v* for comparisons involving pairs of nominal variables. Effect sizes for Cohen’s *d* are conventionally classified as small (*d* = 0.20), medium (*d* = 0.50), and large (*d* = 0.80) [44]. To give precision to these classifications for values of *d* falling between these values, we classified *d* in terms of ranges, using the midpoint between 0.20 and 0.50, and midpoint between 0.50 and 0.80, so as to distinguish among small, medium, and large values of *d*; that is, small 0.20–0.349, medium 0.35–0.649, and large > 0.65. The corresponding criteria for classifying Cramér’s *v* and the range for interpreting scores were as follows: small (0.1, 0.1–0.19), medium (0.3, 0.2–0.39), and large (0.5, ≥ 0.40).

## Results

### Structure of declining adherence to SDIS

Exploratory factor analysis of the SDIS items indicated a single-factor solution with only the first Eigen value being greater than 1.00. The first 5 Eigen values were 3.98, 0.98, 0.80, 0.74, and 0.69. Factor loadings are shown in Table 2, indicating that all loadings were salient (> 0.30), indicating that all items strongly loaded on the factor. The SDIS scale, representing the sum of the 10 SDIS items, had a high internal consistency (Table 1). The total score was used as the input variable for the Latent Class Analyses. The results, shown in Table 4, indicated that the best fitting model consisted of two classes; Class 1 (92% of sample, *n* = 5,326), Class 2 (8%, *n* = 486). Scores on the total score were higher in Class 1 (M = 0.2, SD = 0.5) than Class 2 (M = 4.7, SD = 1.8), *t*(5,810) = 134.14, *p* < .001. This indicates that declining adherence was significantly greater for Class 2 than Class 1. The mean score for Class 1 was close to zero, indicating little change in SDIS adherence. The results show that the majority of participants were largely adherent to SDIS, but a sizeable minority (8%) reported a deterioration in SDIS, indicative of pandemic fatigue.

**Table 4:** Fit indices for Latent Class Analysis.

No. classes	Akaike Information Criterion	Bayesian Information Criterion	Sample size adjusted Bayesian Information Criterion	Bootstrap likelihood ratio difference test χ^2^(*df =* 2)	Bootstrap likelihood ratio difference test *p*
**1**	20,761.93	20,775.26	20,768.91	--	--
**2**	**15,878.47**	**15,905.15**	**15,892.43**	**4,887.45**	< 0.001
**3**	15,882.47	15,922.48	15,903.41	< 0.01	> 0.999

Bold = best-fitting model; -- = not applicable.

### Comparison of adherent and non-adherent groups

Table 5 shows the comparisons between classes on a range of personality, emotion-related, and COVID-19-related variables. Statistical power to detect small effect sizes at α = 0.01 was 0.95 for these analyses. Accordingly, the study was sufficiently powered to detect even small differences between classes. To facilitate the interpretation of the results, a Discriminant Function Analysis was conducted to determine which variables best distinguished the two classes. Input variables for this analysis were those having small, medium, or large effects in Table 5. The table shows the loadings of the variables on the discriminant function. Salient (>0.30) loadings are in bold. Taking both the effect sizes and discriminant loadings into consideration, Table 5 shows that people in Class 2, compared to Class 1, reported a greater current disregard for SDIS. In other words, for Class 2 there was a progressive past-year deterioration in SDIS as well as a current low level of adherence.

**Table 5:** Comparisons between classes.

Variable	Class 1 (n = 5,326): M (SD) or %	Class 2 (n = 486): M (SD) or %	*t*(*df* = 5,810) or χ(*df* = 1)	*p*	ES	Type of ES	DFA loading
**Disregard for SDIS, current**	2.2 (3.1)	5.2 (4.7)	19.76	< 0.001	0.94^a^	*d*	**0.60**
**Coping with COVID-19**	90.4 (24.1)	113.1 (30.5)	19.44	< 0.001	0.92^a^	*d*	**0.59**
**Stressors associated with COVID-19**	40.4 (15.3)	54.0 (21.8)	18.07	< 0.001	0.86^a^	*d*	**0.54**
**Belief in COVID-19 conspiracy theories**	2.2 (3.2)	4.9 (4.7)	17.42	< 0.001	0.83^a^	*d*	**0.53**
**COVID-19 exaggerated**	3.1 (3.1)	5.4 (3.7)	15.14	< 0.001	0.72^a^	*d*	**0.46**
**COVID-19 apathy**	16.2 (5.6)	19.8 (7.0)	13.12	< 0.001	0.62^b^	*d*	**0.40**
**Robust personal health**	4.5 (2.9)	6.2 (3.1)	12.34	< 0.001	0.59^b^	*d*	**0.37**
**Narcissism**	20.2 (5.8)	23.5 (5.8)	12.14	< 0.001	0.58 ^b^	*d*	**0.37**
**Age (years)**	49.3 (17.0)	40.8 (15.2)	10.70	< 0.001	0.51^b^	*d*	**-0.32**
**Perceived socioeconomic status**	5.7 (1.9)	6.6 (1.9)	10.19	< 0.001	0.48^b^	*d*	**0.31**
**Anxiety**	4.7 (5.6)	7.3 (6.0)	9.66	< 0.001	0.46^b^	*d*	0.29
**Depression**	5.9 (6.7)	9.0 (7.6)	9.57	< 0.001	0.45^b^	*d*	0.29
**Sociability**	16.8 (4.6)	18.7 (3.9)	8.84	< 0.001	0.42^b^	*d*	0.27
**COVID-19 anti-vaccination attitudes, current**	36.6 (12.9)	41.6 (12.9)	8.15	< 0.001	0.39^b^	*d*	0.25
**COVID-19 pessimism**	38.1 (11.6)	42.6 (14.8)	7.96	< 0.001	0.38^b^	*d*	0.24
**Psychological entitlement**	3.6 (1.1)	4.0 (1.3)	7.72	< 0.001	0.37^b^	*d*	0.23
**Agreeableness**	10.4 (2.3)	9.7 (2.3)	7.12	< 0.001	0.34^c^	*d*	-0.21
**Conscientiousness**	11.0 (2.4)	10.3 (2.6)	5.76	< 0.001	0.27^c^	*d*	-0.17
**Extraversion**	7.3 (3.0)	8.0 (2.6)	5.17	< 0.001	0.25^c^	*d*	0.16
**COVID-19 burnout**	29.1 (12.4)	31.8 (12.4)	4.74	< 0.001	0.23^c^	*d*	0.14
**Self-reported diagnosis of COVID-19**	7	28	264.37	< 0.001	0.21^b^	*v*	**0.50**
**Belief that COVID-19 has harmed one’s mental health**	0.7 (0.5)	0.8 (0.4)	3.95	< 0.001	0.19	*d*	--
**Country (1 = U.S., 0 = Canada)**	49	77	137.75	< 0.001	0.15^c^	*v*	**0.36**
**Loneliness**	5.3 (1.9)	5.6 (1.9)	2.78	0.005	0.13	*d*	--
**Political conservatism**	3.6 (1.7)	3.8 (1.9)	1.64	0.101	0.08	*d*	--
**Negative emotionality**	7.0 (1.0)	7.1 (1.0)	1.63	0.102	0.08	*d*	--
**Openness to Experience**	9.9 (2.4)	9.7 (2.4)	1.67	0.095	0.08	*d*	--
**Current mental health diagnosis**	23	26	3.28	0.070	0.07	*v*	--
**Distrust in government**	0.7 (12.2)	1.4 (10.8)	1.19	0.234	0.06	*d*	--
**Personally knew someone diagnosed with COVID-19**	53	64	21.52	< 0.001	0.06	*v*	--
**Prior medical condition**	38	28	22.19	< 0.001	0.06	*v*	--
**Female (1 = female, 0 = other)**	58	48	17.17	< 0.001	0.05	*v*	--
**College (1 = full or partial college, 0 = less than college)**	78	85	16.40	< 0.001	0.05	*v*	--
**Received one or both doses of COVID-19 vaccine**	32	41	15.46	< 0.001	0.05	*v*	--
**Healthcare worker**	4	8	13.16	< 0.001	0.05	*v*	--
**Unemployed (1 = unemployed, 0 = other)**	9	5	7.97	0.005	0.04	*v*	--
**Facemask non-adherence, current**	13.1 (3.6)	13.0 (3.2)	0.77	0.440	0.04	*d*	--
**Single (1 = single, 0 = other)**	47	41	5.73	0.017	0.03	*v*	--
**White (1 = White, 0 = other)**	65	59	6.84	0.009	0.03	*v*	--
**COVID-19 blame**	23.4 (6.8)	23.5 (7.5)	0.48	0.634	0.02	*d*	--

*d* = Cohen’s *d*; *v* = Cramér’s *v;* DFA = Discriminant function analysis for variables with small or larger effect sizes, with salient loadings in bold and -- = not applicable. Effect size (ES): a = large, b = medium, c = small.

Table 5 compares the classes on the variables of focus of the present study (e.g., disregard for SDIS, narcissism, psychological entitlement) as well as comparing the classes on background variables such as demographics. These background variables were included in order to more fully characterize the differences between classes. The classes differed primarily on pandemic fatigue; that is, declining adherence to SDIS combined with burnout and related variables. However, as seen in Table 5, the classes also had particular demographic differences.

People in Class 2, compared to those in Class 1, tended to be younger and perceived themselves as being more affluent (Table 5). There were more Americans in Class 2 (77%) than in Class 1 (49%); that is, more Americans than Canadians tended to be non-adherent to SDIS. People in Class 2 were more likely to believe that the COVID-19 threat was a hoax or exaggerated, even though these individuals were more likely to have reportedly contracted the SARSCOV2 virus. The latter finding is consistent with reports that SARSCOV2 often produces mild illness, particularly among young and healthy individuals [20]. People in Class 2 reported greater stressors related to COVID-19 and also reported greater efforts at attempting to cope with those stressors.

### Reasons for adherence to SDIS

For people who generally adhered to SDIS guidelines (i.e., Class 1), Table 3 shows the participants’ reasons for adherence. Here it can be seen that the most common reasons for adherence were to ensure the safety of oneself (87% of participants), safety of one’s community (86%), safety of loved ones (86%), and safety of one’s country (83%). Altruistic and self-preserving reasons were most common. A substantial proportion of people in Class 1 reported that they adhered to SDIS for fear of some sort of punishment, such as disapproval from other people for non-adherence (51%), receiving a fine (36%), or being publicly shamed for non-adherence (35%). Thus, the results suggest that people in Class 1 adhered to SDIS guidelines because of a mix of positive incentives (e.g., personal safety) and fear of punishment or shaming.

## Discussion

Pandemic fatigue is an important problem for managing COVID-19 [1] and likely to be a salient obstacle in mitigating future pandemics. The present study, conducted during the second year of the COVID-19 pandemic, found that past-year decline in adherence to SDIS had a categorical latent structure, consisting of an SDIS adherent group (Class 1: 92% of the sample) and a group reporting a progressive decline in adherence to SDIS (Class 2: 8% of the sample). Class 2 had features indicative of pandemic fatigue; specifically, in addition to reporting a decline in adherence to SDIS, this group had various features consistent with pandemic-related burnout. Compared to Class 1, Class 2 had greater levels of emotional burnout, pessimism, apathy, and cynical or negative beliefs about the COVID-19 pandemic (e.g., believing COVID-19 to be a hoax). The present study confirmed previous findings that pandemic fatigue is associated with the perception that lockdown is unnecessary and ineffective [13], younger age [14–17], greater perceived personal affluence [18], and lower trust in government [18]. People in Class 2, compared to Class 1, tended to be more narcissistic, entitled, and gregarious, and were more likely to report having been infected with SARSCOV2, which they regarded as an exaggerated threat. In other words, pandemic fatigue was associated with heightened self-interest to the expense of community needs.

People in Class 2 also reported higher levels of pandemic-related stress, anxiety, and depression, and described making active efforts at coping with SDIS restrictions that they perceived as unnecessary and stressful. People in Class 1 generally reported that they engaged in SDIS for the benefit of themselves and their community, although 35% also feared they would be publicly shamed if they did not comply with SDIS guidelines. The findings suggest that pandemic fatigue affects a substantial minority of people and, importantly, that many SDIS-adherent people also experience emotionally adverse effects (i.e., fear of being shamed) related to SDIS.

Knowledge alone is not enough to overcome pandemic fatigue, as previous surveys have demonstrated that most people are knowledgeable about COVID-19 protective behaviors [1]. Consistent with previous surveys [1], we found that the majority of respondents were adherent to SDIS guidelines. During the COVID-19 pandemic, community leaders expressed frustration and dismay at people violating social distancing guidelines, using pejoratives such as “Covidiot” to label these individuals [45]. Although public shaming has long played a role in the regulation of societies and can effectively inhibit some forms of socially disruptive behavior [46], public shaming during a pandemic adds a layer of stress on an already distressed public. The burden of accumulated adversity—the piling up of stressors on an individual—is a risk factor for stress-related disorders such as posttraumatic stress disorder [47]. Accordingly, shaming people who are already pandemic fatigued—experiencing dysphoria, anxiety, and irritability about COVID-19—is likely to worsen their mental health. Community leaders and others in positions of authority are advised to use caution when considering shaming people who are not complying with SDIS or other pandemic mitigation guidelines.

The present study had various strengths and limitations. In terms of strengths, the sample size was large, robust statistical methods were used, and the assessment period was timely, given that pandemic-related restrictions had been in place for over a year. Regarding limitations, the assessment of SDIS was retrospective, based on self-report, and the generalizability of the results across different demographic and geographic groups remains to be investigated. Retrospective and prospective assessments each have their strengths and limitations, and ideally both would be conducted, but this was not possible for logistic reasons. Research suggests that behavioral and self-report measures of SDIS produce broadly similar results [6].

Participants were asked to report on their socially undesirable behaviors (i.e., non-adherence to SDIS) and the question arises as to whether the results were affected by a social desirability bias; that is, the tendency to give socially desirable answers to the assessment battery. It might be argued that Class 2 simply represents a group of people who are more willing to admit to socially undesirable attitudes or acts, such as non-adherence to SDIS. This explanation is unlikely for two reasons. First, responding was anonymous. Second, our previous COVID-19 research found that social desirability was unrelated to a range of behavioral, attitudinal, and emotion variables [23]. Social desirability, as assessed by the Marlowe-Crowne Social Desirability Scale Short Form [48], was uncorrelated (i.e., effect sizes below the threshold for “small”) with scores on various pandemic-related attitudes and behaviors, including disregard for SDIS (*r* = 0.03), self-reported violation of pandemic lockdown (i.e., remain-at-home orders) during early 2020 (*r* = 0.02), belief that the COVID-19 threat is exaggerated (*r* = -0.01), belief in COVID-19 conspiracy theories (*r* = -0.04), and anti-vaccination attitudes in general (*r* = -0.03) (*N*s ranged from 3,314 to 6,854) [24]. Thus, it is unlikely that social desirability affected the results of the present study.

Whether the results of the present study generalize to more protracted, highly restrictive SDIS programs, such as stay-at-home mandates imposed over extended periods of time, remains to be investigated. Under such conditions, the structure of pandemic fatigue, as identified in the present study, may be altered. Non-adherence (as in Class 2) is likely to be found under conditions of more severe lockdowns, unless there are efforts to offset the problem. Pandemic fatigue may also start to appear in people who have been generally adherent (as in Class 1). Future research is also needed to investigate potentially relevant variables that were not examined in the present study. For example, boredom proneness is a trait characterized by the tendency to readily become bored in a wide range of situations [49]. This trait was associated with non-adherence to SDIS early in the COVID-19 pandemic and may play a role in pandemic fatigue [50].

Further research is needed to identify strategies for easing the mental health burden imposed by SDIS. Several studies have found that SDIS harms mental health, with protracted SDIS being correlated with substantial increases in anxiety, depression, substance abuse, and other psychological problems [2]. Humans are inherently social creatures, and SDIS involves thwarting this natural urge to socialize. Moreover, research suggests that narcissism (a feature of Class 2 in the present study) is becoming more prevalent in Western societies, likely due to a range of sociocultural factors [51]. This raises concerns about the future of pandemic mitigation methods such as SDIS, which require people to work for the collective good rather than focusing on individuals needs or desires.

Non-adherence to SDIS occurs more rapidly when more severe restrictions are imposed (e.g., stay-at-home orders) as compared to less restrictive alternatives (e.g., overnight curfews) [52]. This might suggest that less restrictive alternatives should be used, although there are concerns that less restrictive methods may be less effective in stemming the spread of infection [52]. Accordingly, future research is needed to identify the optimal form of SDIS, balancing two competing goals: (a) the need to reduce interpersonal contact in order to stem the spread of infection, versus (b) the need to allow people to live their lives with the least restriction so as to limit the harmful effects of SDIS on mental health.

The WHO described a number of methods intended to reinvigorate people to follow SDIS guidelines; see refs. [1] and [53–55]. The efficacy of such methods remains to be established. Encouraging or “nudging” people to follow the guidelines may have greater impact on people who are already amendable to following SDIS guidelines (i.e., Class 1). For people who are narcissistic and distressed, and who see the pandemic restrictions as unnecessary, nudges may be ineffective. Indeed, during the 1918 Spanish flu and during COVID-19, governments responded to non-adherent individuals by becoming increasingly punitive, such as imposing fines or even arresting people who do not comply with SDIS mandates [2, 50]. Alternatives to SDIS have been considered, such as the controversial Great Barrington Declaration, which advocates that only the elderly and medically compromised should be subject to stay-at-home orders during COVID-19 [56]. This proposal has been widely criticized as discriminatory and likely to result in greater morbidity and mortality than existing SDIS measures [57, 58]. During COVID-19, communities experimented with alternatives such as short-term “circuit breaker” lockdowns, in which lockdown and sometimes curfews were imposed for short periods (e.g., two weeks) to attempt to disrupt the spread of infection. The tolerability and efficacy of this and other alternative methods of SDIS that may have less of an impact on mental health remain to be investigated.
